# Deep learning image segmentation approaches for malignant bone lesions: a systematic review and meta-analysis

**DOI:** 10.3389/fradi.2023.1241651

**Published:** 2023-08-08

**Authors:** Joseph M. Rich, Lokesh N. Bhardwaj, Aman Shah, Krish Gangal, Mohitha S. Rapaka, Assad A. Oberai, Brandon K. K. Fields, George R. Matcuk, Vinay A. Duddalwar

**Affiliations:** ^1^ Keck School of Medicine, University of Southern California, Los Angeles, CA, United States; ^2^ Department of Applied Biostatistics and Epidemiology, University of Southern California, Los Angeles, CA, United States; ^3^ Bridge UnderGrad Science Summer Research Program, Irvington High School, Fremont, CA, United States; ^4^ Department of Biology, University of Texas at Austin, Austin, TX, United States; ^5^ Department of Aerospace and Mechanical Engineering Department, Viterbi School of Engineering, University of Southern California, Los Angeles, CA, United States; ^6^ Department of Radiology & Biomedical Imaging, University of California, San Francisco, San Francisco, CA, United States; ^7^ Department of Radiology, Cedars-Sinai Medical Center, Los Angeles, CA, United States; ^8^ Department of Radiology, Keck School of Medicine of the University of Southern California, Los Angeles, CA, United States; ^9^ Department of Radiology, USC Radiomics Laboratory, Keck School of Medicine, University of Southern California, Los Angeles, CA, United States

**Keywords:** bone cancer, CT, deep learning, image segmentation, MRI, PET/CT

## Abstract

**Introduction:**

Image segmentation is an important process for quantifying characteristics of malignant bone lesions, but this task is challenging and laborious for radiologists. Deep learning has shown promise in automating image segmentation in radiology, including for malignant bone lesions. The purpose of this review is to investigate deep learning-based image segmentation methods for malignant bone lesions on Computed Tomography (CT), Magnetic Resonance Imaging (MRI), and Positron-Emission Tomography/CT (PET/CT).

**Method:**

The literature search of deep learning-based image segmentation of malignant bony lesions on CT and MRI was conducted in PubMed, Embase, Web of Science, and Scopus electronic databases following the guidelines of Preferred Reporting Items for Systematic Reviews and Meta-Analyses (PRISMA). A total of 41 original articles published between February 2017 and March 2023 were included in the review.

**Results:**

The majority of papers studied MRI, followed by CT, PET/CT, and PET/MRI. There was relatively even distribution of papers studying primary vs. secondary malignancies, as well as utilizing 3-dimensional vs. 2-dimensional data. Many papers utilize custom built models as a modification or variation of U-Net. The most common metric for evaluation was the dice similarity coefficient (DSC). Most models achieved a DSC above 0.6, with medians for all imaging modalities between 0.85–0.9.

**Discussion:**

Deep learning methods show promising ability to segment malignant osseous lesions on CT, MRI, and PET/CT. Some strategies which are commonly applied to help improve performance include data augmentation, utilization of large public datasets, preprocessing including denoising and cropping, and U-Net architecture modification. Future directions include overcoming dataset and annotation homogeneity and generalizing for clinical applicability.

## 1. Introduction

Bone is the third most common site of metastasis in the human body across all cancers, with an incidence of 18.8 cases per 100,000 each year and survival rates ranging from months to a few years (1, 2). The most common origins of bone metastases include breast, prostate, lung, and hematologic malignancies (1). Primary bone sarcomas are uncommon, with an incidence of 0.9 cases per 100,000 each year and higher survival rate (3).

**Figure 1:**
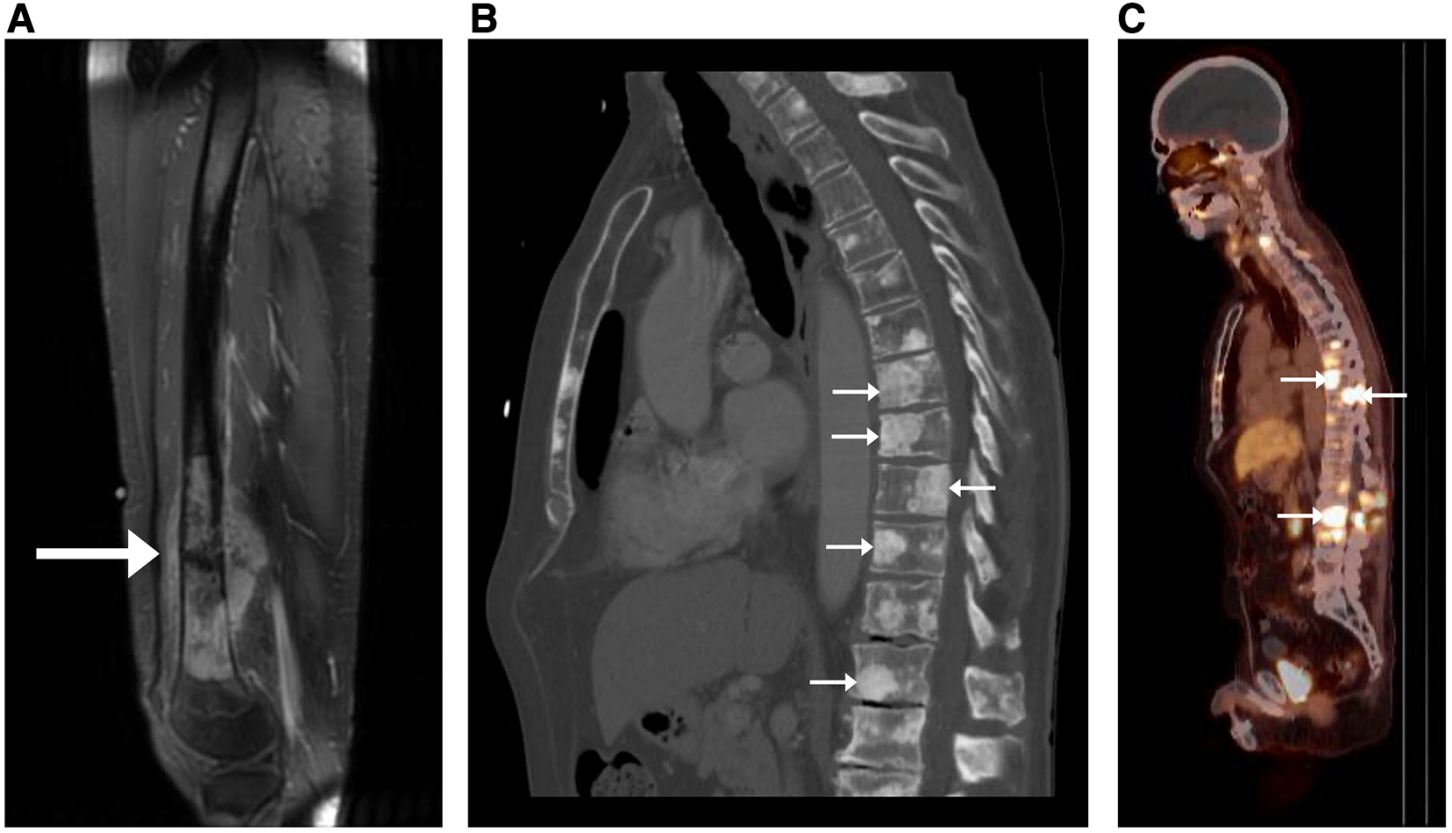
Appearance of malignant bone lesions on different imaging modalities. (**A**) Sagittal T1-weighted post-contrast MR image with fat suppression of the right femur in a 32-year-old female with biopsy-proven osteosarcoma of the distal femoral metadiaphysis (arrow). (**B**) Sagittal chest CT with bone windows showing diffuse osseous metastatic disease (arrows) in a 72-year-old male with castration-resistant prostate cancer. (**C**) Sagittal vertex-to-pelvis prostate-specific membrane antigen (PSMA) PET/CT fusion image showing diffuse osseous metastatic disease (arrows) in the same patient an in (**B**) 6 months previously. Note that in (**Β**) and (**C**), not all metastatic lesions have been annotated with arrows.

Magnetic Resonance Imaging (MRI), Computed Tomography (CT), and Positron-Emission Tomography/CT (PET/CT) are commonly used to diagnose and track malignant bone lesions (Figure 1). MRI has higher sensitivity to detecting lesions in both the marrow and surrounding soft tissue structures and does not expose the patient to ionizing radiation. However, MRI requires a more expensive and laborious imaging process when compared with CT (4). CT is more sensitive to detecting changes in bone morphology and has higher spatial resolution, although it involves radiation and has poorer performance with soft-tissue and marrow imaging (5). PET/CT combines techniques of both CT (three-dimensional x-ray scanning with high spatial resolution) and PET (injection of radioactive tracer to quantify cellular metabolism), providing high sensitivity and specificity for imaging skeletal malignancies (6). These benefits make PET/CT the standard of care in bone lesion imaging, although there are still the drawbacks of higher cost and use of radiation. PET/MRI similarly offers combined benefits of both MRI and PET. Malignant bone lesions often appear as blastic (hyperdense regions indicating bone formation), lytic (hypodense regions indicating bone resorption), or a mix.

Early diagnosis of malignant bone lesions is critical for improving prognosis and treatment response. Image segmentation, in which the boundaries of a lesion are precisely delineated, allows radiologists to determine the extent of disease and accurately provide quantitative measurement for disease tracking, treatment response, and management (7). Additionally, accurate segmentation is essential for performing clinical research using radiologic images. The task of image segmentation is typically performed manually by radiologists, but this is a labor-intensive and time-consuming process, thus limiting its applicability in clinical workflows.

**Figure 2:**
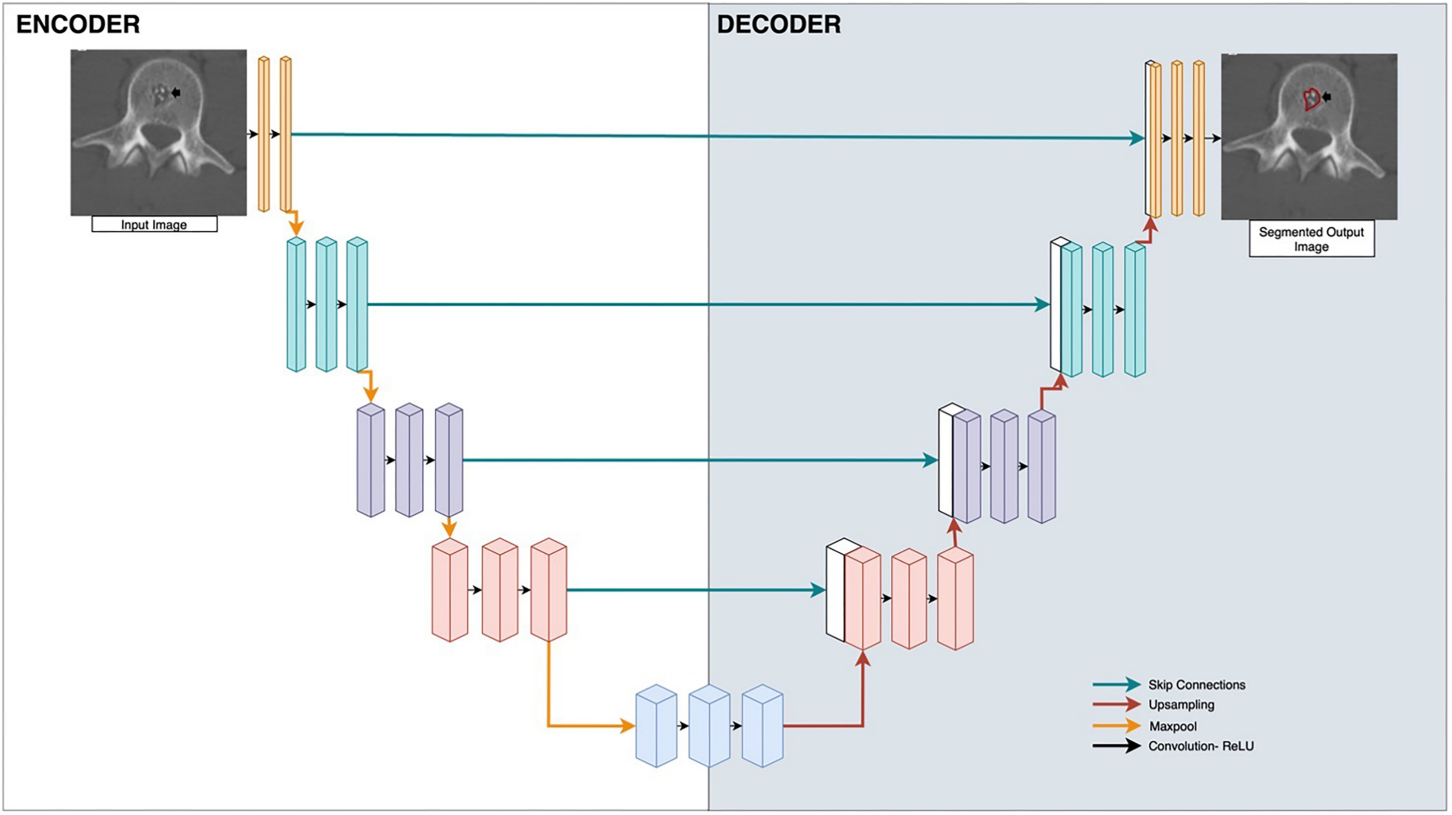
U-Net applied to bone radiology image segmentation. Input is the medical image, and output is the segmentation mask applied to the lesion. Boxes represent vectorized outputs of convolutional and pooling operations. Arrows represent mathematical operations applied to each layer. Blue arrows are skip connections, red arrows are upsampling, yellow arrows are maxpool, black arrows are Convolution-rectified linear units (ReLU).

Machine learning has the potential to automate lesion segmentation. Some early image segmentation methods include thresholding, region-growing, edge-based segmentation, active contour models, watershed transforms, and snakes (8). All of these methods involve identifying simple features of an image such as thresholded intensity values, edges, or neighboring homogeneous regions, but are limited in analyzing more complex features (9). The progress of deep learning methods in particular, especially Convolutional Neural Networks (CNNs) (10), provides the ability to segment complex images with increasing accuracy (8, 11, 12). CNNs are deep neural networks in which convolution operations are applied as sliding filters over an image, reducing dimensionality, and identifying image features through selection of filter weights. A particularly popular CNN architecture is U-Net, which consists of an initial encoding section of convolution operations and a subsequent decoding section of transpose-convolution operations to reconstruct an image with the same dimensions as the input (13) (Figure 2). Deep learning has shown promise in image segmentation of lesions in CT and MRI scans in a wide range of contexts including lesions of the breast (14), kidney (15), and brain (16, 17).

Deep learning model performance generally improves with larger dataset sizes, with the minimal acceptable size typically being on the order of hundreds of subjects. However, this is a challenging task in the realm of medicine where the input involves patient data due to concerns regarding privacy and sharing (18). While there are some major public databases that can assist with data augmentation or transfer learning for certain clinical queries (19–22), there are many pathologies that are specific or unique enough where such datasets are not readily available. Some techniques to try to overcome this deficit include working with large pretrained models (23), data-generation techniques such as Generative Adversarial Networks (14, 24, 25), or applying domain knowledge to data preprocessing and augmentation (26, 27). There are very few public datasets or models which capture primary or metastatic skeletal lesions on CT, MRI, PET/CT, or PET/MRI.

The purpose of this systematic review and meta analysis is to describe how effective deep learning-guided image segmentation techniques are in accurately identifying and delineating malignant bone lesion on major radiologic imaging studies (CT, MRI, PET/CT, and PET/MRI), as well as to compare methods and performance across studies. We describe all algorithms and neural network architectures reported in the included studies, as well as characteristics of the datasets and additional techniques used for successful segmentation. We also note any publicly available datasets or models.

## 2. Materials and methods

### 2.1. Literature search

Our systematic literature review is in compliance with the guidelines outlined by the Preferred Reporting Items for Systematic Reviews and Meta-Analyses 2020 (PRISMA). We performed a keyword search for papers which studied deep learning-based image segmentation of cancerous lesions of the bone on CT, MRI, PET/CT, and PET/MRI scans. Searches were performed on Pubmed, Embase, Web of Science, and Scopus. All searches were performed on May 8, 2023. The exact search criteria were as follows:

“(CT OR CTs OR MRI OR “MR Imaging” OR “PET-CT” OR “PET/CT” OR “PET-MRI” OR “PET/MRI”) AND (Segmentation) AND (“machine learning” OR “deep learning” OR “artificial intelligence” OR “neural network” OR “neural networks” OR “auto-segmentation” OR “auto segmentation”) AND (bone OR skeleton OR bones OR osseous OR blastic OR lytic) AND (cancer OR cancers OR metastases OR metastasis OR neoplasm OR neoplasms OR metastatic OR tumor OR tumors OR malignant OR tumour OR tumours)”

Other inclusion criteria included a publication date range of 2010–2023, use of English language, full text availability, and only primary literature (i.e., other review articles were excluded). Exclusion criteria included segmentations performed on other imaging modalities (e.g., x-ray, bone scintigraphy, PET), other types of tissues or organs, segmentation of non-malignant features (e.g., whole bone segmentation, fracture segmentation), and non-segmentation techniques (e.g., synthetic data creation, boundary-box generation, outcome classification).

We used the Covidence platform for paper importing and screening (28). All unique papers which fit these criteria were passed through a primary screening of titles and abstracts by a single reviewer. All papers which passed the primary screen were then passed through a secondary screen involving full text review for inclusion criteria by two reviewers.

### 2.2. Data extraction

Categories for data extraction were chosen to describe imaging modality, model type, dataset, lesion type, and part of body in more detail. Data was extracted from each paper with the following categories (Supplementary Table S1):

 (1) Publication date
 (2) Imaging modality (CT, MRI, PET/CT, or PET/MRI)
 (3) Imaging dimensionality (2-Dimensional [2D], 3-Dimensional [3D])
 (4) Primary cancer type
 (5) Quality of lesion (blastic, lytic, or mixed)
 (6) Soft tissue component
 (7) Model architecture
 (8) Dataset publicity
 (9) Dataset size (patients, images)
 (10) Patient population (demographics)
 (11) Treatment received
 (12) Ground truth establishment
 (13) Training-cross validation-test split
 (14) Cross validation method
 (15) Additional methods
 (16) Metrics.

## 3. Results

### 3.1. PRISMA flowchart

**Figure 3:**
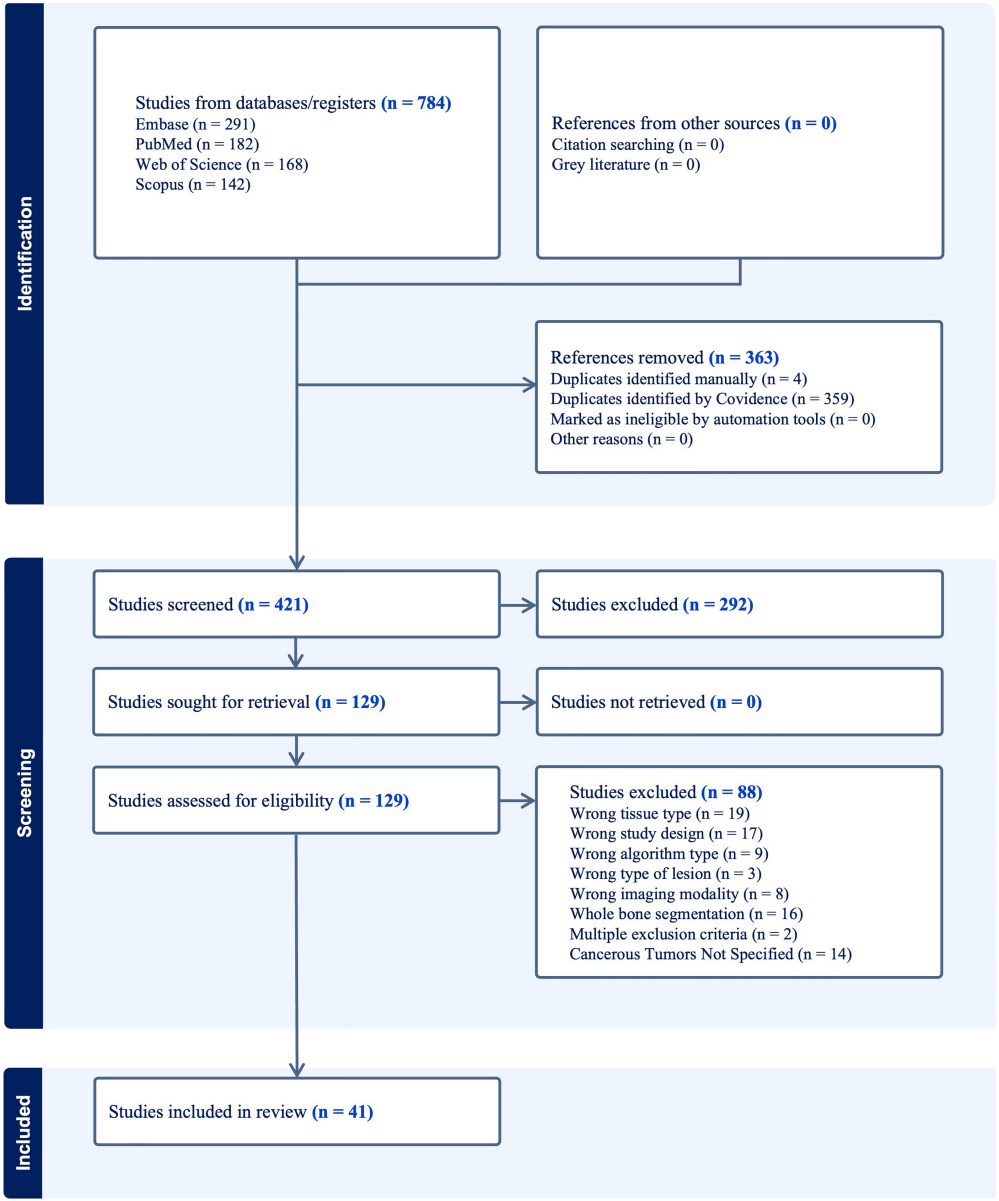
PRISMA flowchart of systematic literature review.

The results of our literature search are shown in the PRISMA flowchart (Figure 3). In brief, our initial search yielded 784 papers. Covidence automatically eliminated 363 duplicates. An additional 4 duplicates were eliminated manually, leaving 421 unique manuscripts. After primary screening of titles and abstracts, 292 papers were further excluded. From the 129 papers which passed through full-text review, 41 studies were ultimately eligible for inclusion in this study (Supplementary Table S1) (29–69). Some of the most common reasons for exclusion included wrong tissue type, segmentation of a non-malignant feature (e.g., whole bone segmentation or fracture segmentation), wrong study design (e.g., prognosis classification, boundary box), and wrong imaging modality (e.g., bone scintigraphy, PET, x-ray).

### 3.2. Categorization of included studies

**Figure 4:**
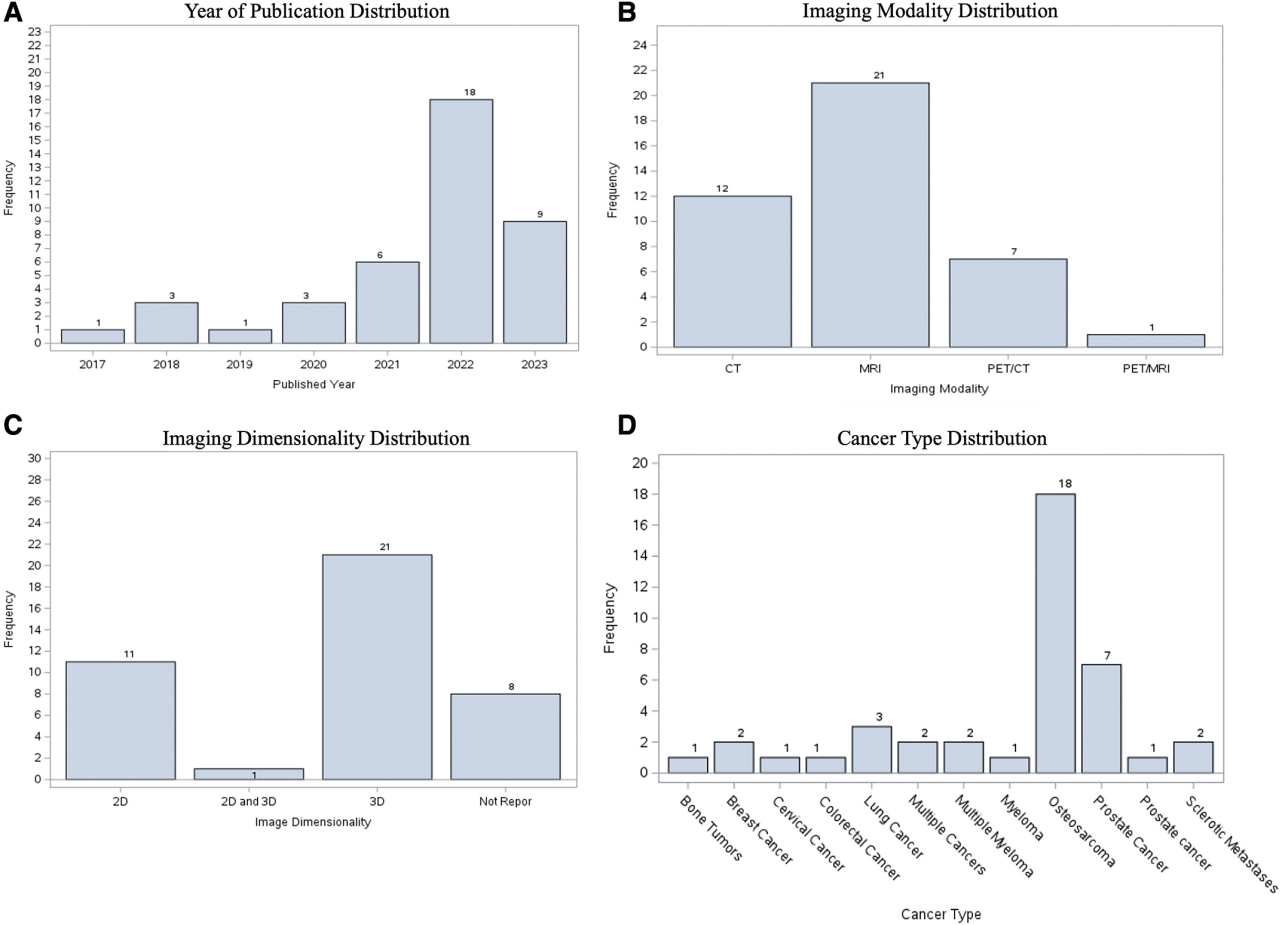
Visualization of characteristics of included studies, showing distribution according to (**A**) publication year; (**B**) imaging modality; (**C**) image dimensionality; (**D**) type of cancer.

Of the 41 total studies, the most popular publication year was 2022 (*n* = 18 studies, 43.90% of the cohort), followed by 2023 (up until May) (*n* = 9, 21.95%). While our search criteria ranged from 2010, the oldest paper included was from 2017. The most common imaging modality studied was MRI (*n* = 21, 51.22%), followed by CT (*n* = 12, 29.27%). The most common image dimensionality method used 3D data alone (*n* = 21, 51.22%), followed by 2D alone (*n* = 11, 26.83%). Osteosarcoma was the most common cancer primary bone malignancy (*n* = 18, 43.90%). Prostate cancer was the most secondary bone malignancy (*n* = 7, 17.07%) (Figure 4).

### 3.3. Synthesized findings of included studies

**Figure 5:**
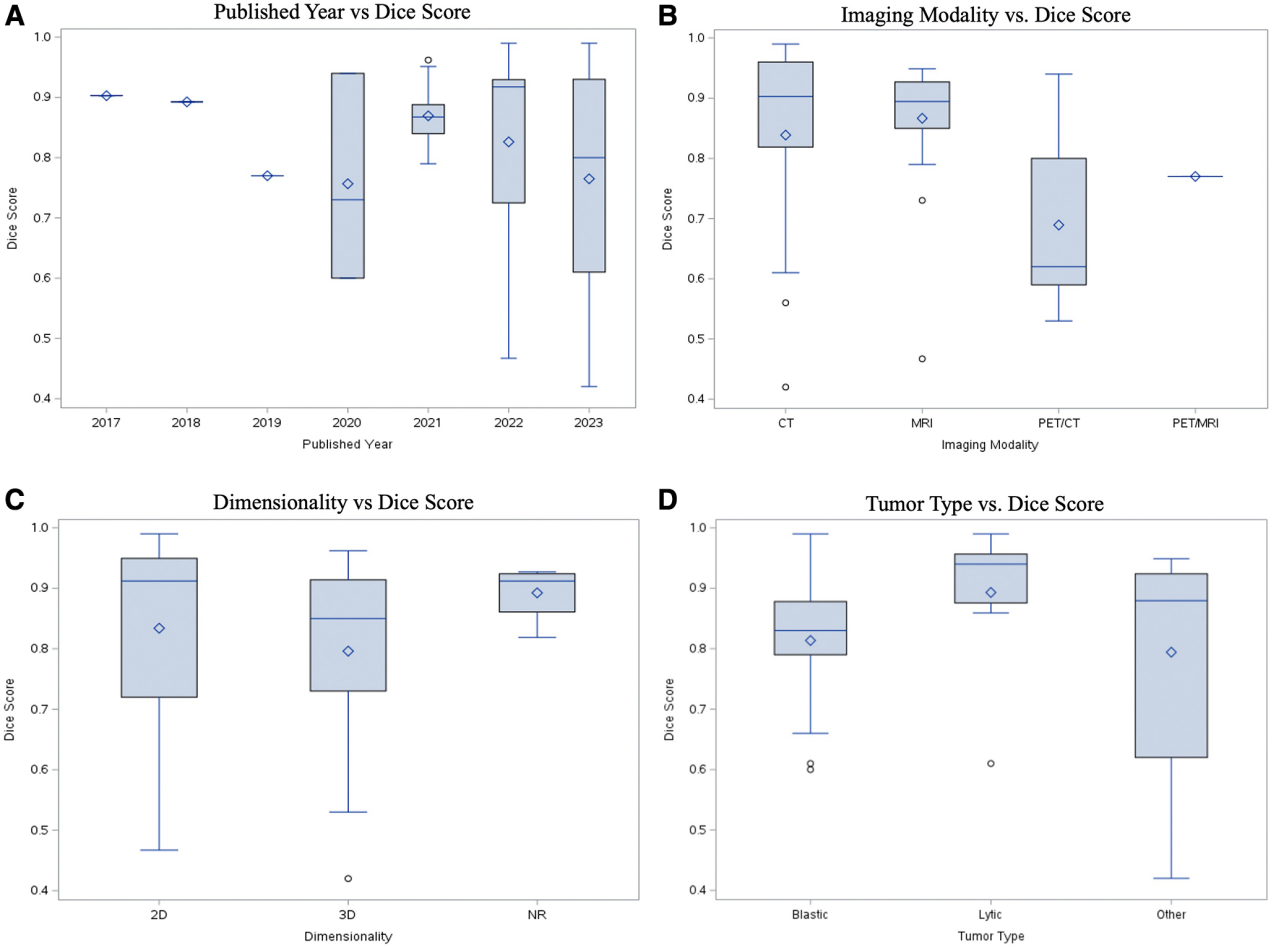
Performance comparison with DSC by (**A**) publication year; (**B**) imaging modality; (**C**) image dimensionality; (**D**) quality of lesion (blastic vs. lytic).

Studies were categorized primarily by dimensionality, modality, publication year, and lesion characteristics (i.e., blastic vs. lytic). All performance metrics reported by each paper, including dice similarity coefficient (DSC), F1-measure, Jaccard, accuracy, sensitivity, and specificity, were included in Supplementary Table S1. DSC was by far the most popular metric, recorded in 35 papers (85.3%). In order to determine statistical significance between groups, a simple two-sample t test was conducted with a power level of 95% being established prior to analysis. While there was a higher median DSC for studies which used 2D data (0.901, *n* = 11) compared to 3D data (0.856, *n* = 17), the difference was not statistically significant (Figure 5C, Supplementary Table S2). In the years 2017 through 2019, there was only a single paper published each year across the 3 years, which reported both the dimensionality method used and a DSC. Although the years 2022 and 2023 accounted for a majority of the papers within the cohort (*n* = 27, 65.85%), there was no statistically significant difference in median DSCs (Figure 5A). With regards to image modality, studies utilizing CT imaging generally reported higher median 2D DSCs (0.94, *n* = 4) compared to MRI (0.924, *n* = 7). In contrast, MRI generally yielded a higher 3D DSCs (0.895, *n* = 10) than studies which evaluated 3D data by CT (0.856, *n* = 5) (Supplementary Table S2). However, neither difference for 2D vs. 3D data was statistically significant (Figure 5C). Aggregating all data dimensionality, CT had a slightly higher median DSC (0.92, *n* = 9) than MRI (0.85, *n* = 17); however, there was no statistically significant difference in mean dice score between the two imaging methods (*p* = 0.5469). Papers studying lytic lesions reported higher median 2D and 3D DSCs, at 0.94 (*n* = 2) and 0.922 (*n* = 5), respectively, when compared to segmentation of blastic lesions, though this difference was similarly not statistically significant (Figure 5D, Supplementary Table S2). Papers which did not include cross-validation showed an average higher DSC (0.923, *n* = 13) than those which did (0.840, *n* = 22) (*p* = 0.0038). There was no statistically significant relationship between using data augmentation in workflow and increased DSC (*p* = 0.1156).

## 4. Discussion

In this systematic review and meta-analysis, we have attempted to aggregate the literature describing automated segmentation methods for primary and metastatic bone malignancies on CT and MRI. We found that most models achieved objectively good performance (DSC >0.7) on this task, with some of the most common methods including data augmentation, U-Net architecture modification, and preprocessing to reduce noise. We clarify the frequency of reported studies that fall into specific criteria regarding imaging approaches and lesion quality, which helps identify which problems still need to be most studied and how much precedent work exists for a specific type of problem. Overall, while small numerical differences were seen between segmentation DSCs when comparing across imaging modality, publication year, dataset dimensionality, and lesion quality (blastic vs. lytic), none of these were found to be statistically significant. The similarity in performance across these attributes indicates that these segmentation models have the capability to perform well across a range of conditions. The statistical significance in DSC improvement for papers which excluded cross validation compared to those which included it indicates the potential of an overfitting problem in these cases, highlighting the importance of test sets and external validation for generalizability. While other reviews have investigated similar segmentation performance tasks applied to various lesions or whole organs, to the best of our knowledge, ours is the first to focus on deep learning techniques applied specifically to lesions of the bone (70–77). Additionally, ours is the first which specifically evaluates differences in segmentation performance specifically as they relate to imaging modality, imaging dimensionality, and predominant lesion characteristic. Future directions include comparing further characteristics of papers (e.g., model architecture, type of cancer, dataset size, etc.) to determine which types of problems or approaches yield the best results, as well as expanding the scope of analysis to other imaging modalities or targets of segmentation to increase statistical power.

### 4.1. Metrics

Comparison of metrics across various studies can be difficult. Different problems or datasets may possess inherently different technical challenges even when problems appear similar, making performance comparison with metrics across studies difficult. Additionally, different metrics capture different qualities of success (Table 1). For instance, specificity is high when there are minimal false positives (i.e., minimal areas of predicted lesions where none is present); since most lesions make up a small percentage of an image, an algorithm will achieve high specificity by predicting no lesions on an image, even though this requires no learning. Within our cohort, Zhao et al. reported an estimated DSC of 0.60, which is considerably lower than most DSCs which lie approximately within the 0.85–0.95 range (69). However, they also reported sensitivity and precision to each be 0.99, which would indicate an element of good performance. While each metric has its strengths and limitations, DSC was the most commonly reported metric by far, reported in nearly every included study. DSC's ubiquity in image segmentation is due to a few factors including its use by many others studying image segmentation techniques, its balance of precision and recall, its intuitive appeal as an approximator of percentage of overlap between ground truth and prediction, its history of being used for measuring reproducibility of manual segmentation, and its adaptability to logit transformation since its values lie between 0 and 1 (78–81). All reported metrics from each study were recorded in Supplementary Table S1. While a uniform dataset-agnostic success criterion cannot be established as a result of the challenges described earlier, a general objective threshold for what is considered a reasonable model is to achieve a DSC around 0.7 (80), which most papers in this review surpass.

**Table 1:** Popular metrics for image segmentation.

Metric	Equation
Dice similarity coefficient	$\frac{\left|P\cap L\right|}{\left|P|+|L\right|}=\frac{2\mathrm{TP}}{2\mathrm{TP}+\mathrm{FP}+\mathrm{FN}}$
F1-measure	$\frac{2*\mathrm{precision}*\mathrm{recall}\;}{\mathrm{precision}+\mathrm{recall}}$
Jaccard index, intersection over union	$\frac{\left|P\cap L\right|}{\left|P|\cup |L\right|}=\frac{\mathrm{TP}}{\mathrm{TP}+\mathrm{FP}+\mathrm{FN}}$
Accuracy	$\frac{\mathrm{TP}+\mathrm{TN}}{\mathrm{TP}+\mathrm{TN}+\mathrm{FP}+\mathrm{FN}}$
Sensitivity, true positive rate, recall	$\frac{\mathrm{TP}}{\mathrm{TP}+\mathrm{FN}}$
Specificity, true negative rate	$\frac{\mathrm{TN}}{\mathrm{FP}+\mathrm{TN}}$
False positive rate	1−specificity
False negative rate	1−sensitivity
Precision, positive predictive value	$\frac{\mathrm{TP}}{\mathrm{TP}+\mathrm{FP}}$
Negative predictive value	$\frac{\mathrm{TP}}{\mathrm{TN}+\mathrm{FP}}$
Area under curve (AUC)	∫(ReceiverOperatingCharacteristicCurve)

P, Prediction; L, Label; TP, true positive; TN, true negative; FP, false positive; FN, false negative.

### 4.2. Imaging modality and dimensionality

The overwhelming majority of imaging modalities utilized throughout the paper cohort were either CT or MRI. Both CT and MRI are reasonably amenable to automated segmentation, with median DSCs between 0.85–0.95 for both modalities (Figure 5B, Supplementary Table S2). Models analyzing PET/CT and PET/MRI data demonstrate lower median DSCs than CT and MRI-trained models. PET/CT and PET/MRI combine spatial and metabolic information, providing useful context for radiologists. However, there can be noise in radioactive tracer uptake involved in PET, and errors in spatial alignment of the two scans, making data more difficult to train (82). Additionally, malignant lesions display heterogeneous metabolic activity, adding further noise to the imaging process. In order to overcome this, Hwang et al. utilized maximum-likelihood reconstruction of activity and attenuation (MLAA) algorithm as input for a CNN to improve accuracy and convergence with good results (40).

Models were able to perform well on both 2D and 3D data, with 2D data achieving slightly higher median DSCs (Figure 5C), although the results were notably not statistically significant. Both types of dimensionalities have pros and cons. Computer vision models were historically trained with two-dimensional images, and 2D data is inherently generally less complex than 3D. However, given that radiologists almost always rely on 3D data for image interpretation, modern deep learning frameworks in radiology, such as nnU-Net (11), have been developed to primarily evaluate with 3D data. The third dimension adds additional spatial and contextual information that may otherwise be lost in two dimensional analysis. As a compromise, one model in our dataset utilized 2.5D data by employing two 2D encoder-decoder modules and one pseudo-3D fusion module, which extracted features from the 2D outputs (53). For clinical applications with unknown cases, considerations for determining data dimensionality for a model include spatial and contextual information, model choice, and difficulty of the segmentation problem.

### 4.3. Dataset size

Dataset size ranged drastically among included papers, with image count ranging from 37 (54) to 80,000 + . Generally, most papers included dataset sizes in the hundreds to low thousands of images or scans. Most studies utilized private and relatively small datasets, making generalizability of algorithms difficult. However, the one large publicly available dataset containing over 80,000 MRI scans of osteosarcoma was utilized by numerous studies (43, 45, 52, 55, 59–64). Dataset size was not a significant predictor of model performance in our cohort, as most models achieved DSCs above 0.7, and many above 0.9, at all ranges of dataset sizes.

This good performance in spite of small dataset size could be attributed in part to data augmentation techniques utilized by many papers. Some of the most popular employed techniques include random cropping, flipping, rotation, zooming, and mirroring (30–32, 35, 38, 43, 50, 52, 54, 56, 60, 67, 68). Of the 14 additional methods found within our review, 7 involved some form of data augmentation. However, as described earlier, there was no correlation between data augmentation workflow and DSC.

Transfer learning was utilized in some cases. Transfer learning is generally thought to be most effective when the transferred data is large and similar to the pathology being studied. Due to the limited nature of public radiology images, models trained on very large datasets of non-radiologic images, such as Microsoft Coco (83), may be reasonable candidates for transfer learning even for image analysis in radiology (66). Similarly, other studies utilized generative methods to create phantom images for their training sets that resembled real images (65). Data preprocessing can incorporate steps to improve model performance, such as whole-bone segmentation to allow the algorithm to have a smaller region to analyze when segmenting an osseous lesion (47).

With small datasets comes the increased risk of overfitting. There was no consensus on training-cross validation-test splits. Generally, most studies dedicated approximately 60%–80% of data to the training set, 10%–30% of data to the test set, and 0%–20% to the cross-validation set (Supplementary Table S1). Nearly half of all papers did not include a cross validation set, meaning that any hyperparameter tuning or architecture adjustment that resulted from testing could have resulted in overfitting. The higher average DSC of papers without cross-validation (0.92) compared to those with it (0.79) supports the likelihood of overfitting in some of these cases. Only two papers utilized external validation (testing of the model on an additional dataset acquired separately from other sets used to initially train or evaluate the model), making generalizability especially difficult (47, 48). However, for both papers, the DSC on the external validation set was the same as that of the test set (at 0.79 and 0.84, respectively), demonstrating model generalizability in these cases (47, 48).

### 4.4. Model architecture

Most studies employed a U-Net CNN architecture for automated image segmentation. U-Net is a popular architecture type because of its ability to accurately segment small targets and fast training speed (84). Image segmentation, as opposed to classification, is especially helpful for extracting objects of interest. In particular, bone segmentation of lesions correctly identifies the spatial location of a tumor. What distinguishes U-Net from other CNNs are the encoder-decoder networks as well as the implementation of skip connectors. The encoder-decoder network ensures that the output image has the same dimensionality as the input image while skip connections ensure full recovery of details and features that may have been lost or forgotten as information passes through successive layers. This preservation of dimensionality is essential for image segmentation, where the output is a binary mask which must resemble the outlined feature on the input image (84). Another attractive feature of the U-Net is the fact that each layer of the network extracts features from a different spatial scale of the image, and by collecting results from each of these layers, the network is able to transform an input image at multiple spatial scales.

Many modifications of U-Net were created to boost model performance. For instance, dilated convolutional U-Net, which involves multiple dilated convolutions following a standard convolution, was employed in a modified U-Net with recurrent nodes in order to preserve contextual information and spatial resolution (36). Some models employed combinations of transformer models and modified U-Nets, allowing for preservation of contextual features such as edge enhancement (45, 49). Cascaded 3D U-Net likewise employ two U-Net architectures in series, with the first trained on down-sampled images and the second trained on full-resolution images, allowing for a combination of granularity and refinement of the features of choice (39).

While a majority of the papers utilized a modification of the U-Net segmentation algorithm, other alternative architectures included non-convolutional Artificial Neural Network models (41), voxel-wise classification (33), AdaBoost algorithms and Chan-Vese algorithms (37), CNN with bagging and boosting (44), and V-Net (34, 65). These alternative algorithms achieved DSCs or AUCs above 0.7, which is on par with the median performance of the U-Net models. However, U-Net variations have been tried in a greater number of studies and demonstrated performance as high as 0.9821 in this cohort (58), indicating that U-Net may be more suitable at present day for achieving maximal performance.

### 4.5. Approaches to segmentation

Two approaches to delineating or segmenting regions of interest are “filling in the lesion” and “tracing precise contour”. Filling in the lesion involves segmenting the entire volume of the region of interest including both the solid and necrotic components of the lesion. On the other hand, tracing precise contours involves precisely outlining the boundaries of a region of interest such that healthy tissues and other non-relevant features are excluded. While the overwhelming number of publications use lesion segmentation as the only methodology, a few studies in literature have discussed a multi step strategy “identification of lesions”, viz creating bounding boxes around the lesions as a separate first step and then a subsequent strategy of precise segmentation of lesions (85, 86). Despite the different implications of these approaches, most papers did not specify which approach they followed when establishing ground truth. If establishment of ground truth was discussed at all, it was usually generally stated the number and skill level of radiologists involved in the process, but with no specific mention of methodology. Even so, Trägårdh et al. studied the importance of inter-reader heterogeneity by comparing model performance on a test set annotated by the same physician who annotated the training set as compared to separate annotators, finding substantial performance differences between sensitivities (57). Methodology of producing ground truth segmentations warrants further discussion to establish a repeatable standard in future studies. The inter-reader heterogeneity also points to the benefit of using probabilistic segmentation algorithms that would account for this variability and produce an ensemble of likely segmentations for a given input image. While these algorithms have been used for the segmentation tasks (17, 87), they have not yet been applied to bone segmentation.

One of the strengths of this review is the comprehensive analysis of all papers fitting search criteria, and the detailed data extraction to allow for comparison of methods or qualities among all papers which have studied this type of problem. Another strength is maintaining focus on clinically relevant features of model design while also keeping in mind technical details of model implementation. A limitation is the difficulty in comparing metrics across studies. Dataset quality, annotation heterogeneity, and noise can make evaluation of a good DSC specific to the specific dataset being studied. Additionally, the relatively small number of studies involved in the review made it difficult to perform any rigorous statistical analysis between subcategories.

In conclusion, deep learning shows great promise for bone lesion segmentation. Considerations include model architecture, imaging modality and dimensionality, dataset size, and establishment of ground truth. Compared to other tissues and organs, there is still much to be done to expand on the task of bone lesion segmentation. Future directions include training on larger and more diverse datasets, applying multiple methods of establishing ground truth, accounting for variability in the segmentation task, and integrating into clinical application. The success with the osteosarcoma MRI dataset from Second Xiangya Hospital of Central South University shows the importance and applicability of these large public datasets (63), and similar efforts should be undertaken from other institutions and studying other types of lesions. General image segmentation models, such as the Segment Anything Model (12), could also show promise in bone lesion segmentation, especially in conjunction with optimization processes involved in the architecture design of these studies. Deep learning-guided segmentation results have great potential to augment human performance, especially in conjunction with radiomic and pathomic data. As these models continue demonstrating success and generalizability, they will help radiologists save time and improve accuracy in delineating these lesions.

## Data Availability

The original contributions presented in the study are included in the article/**Supplementary Material**, further inquiries can be directed to the corresponding author.

## References

[1] Jayarangaiah A Kemp AK Theetha Kariyanna P. Bone metastasis [Updated 2022 Oct 25]. In: StatPearls *[Internet]*. Treasure Island (FL): StatPearls Publishing (2023). Available at: http://www.ncbi.nlm.nih.gov/books/NBK507911/ (Cited Jun 5, 2023). 29939688

[2] Ryan C Stoltzfus KC Horn S Chen H Louie AV Lehrer EJ Epidemiology of bone metastases. Bone. (2022) 158:115783. 10.1016/j.bone.2020.115783 33276151

[3] Franchi A. Epidemiology and classification of bone tumors. Clin Cases Miner Bone Metab. (2012) 9(2):92–5. PMID: 23087718; PMCID: PMC3476517. 23087718 PMC3476517

[4] van Beek EJ Hoffman EA. Functional imaging: CT and MRI. Clin Chest Med. (2008) 29(1):195. vii. 10.1016/j.ccm.2007.12.003 18267192 PMC2435287

[5] Heindel W Gübitz R Vieth V Weckesser M Schober O Schäfers M. The diagnostic imaging of bone metastases. Dtsch Ärztebl Int. (2014) 111(44):741–7. 10.3238/arztebl.2014.0741 25412631 PMC4239579

[6] Kosmin M Padhani AR Gogbashian A Woolf D Ah-See ML Ostler P Comparison of whole-body MRI, CT, and bone scintigraphy for response evaluation of cancer therapeutics in metastatic breast cancer to bone. Radiology. (2020) 297(3):622–9. 10.1148/radiol.2020192683 33078998

[7] Suetens P Bellon E Vandermeulen D Smet M Marchal G Nuyts J Image segmentation: methods and applications in diagnostic radiology and nuclear medicine. Eur J Radiol. (1993) 17(1):14–21. 10.1016/0720-048X(93)90023-G 8348907

[8] Minaee S Boykov Y Porikli F Plaza A Kehtarnavaz N Terzopoulos D. Image Segmentation Using Deep Learning: A Survey. *arXiv*. (2020). Available at: http://arxiv.org/abs/2001.05566 (Cited Jun 5, 2023). 10.1109/TPAMI.2021.3059968 33596172

[9] Luo D Zeng W Chen J Tang W. Deep learning for automatic image segmentation in stomatology and its clinical application. Front Med Technol. (2021) 3:767836. 10.3389/fmedt.2021.767836 35047964 PMC8757832

[10] O'Shea K Nash R. An Introduction to Convolutional Neural Networks. *arXiv*. (2015). Available at: http://arxiv.org/abs/1511.08458 (Cited Jun 6, 2023).

[11] Isensee F Jaeger PF Kohl SAA Petersen J Maier-Hein KH. nnU-Net: a self-configuring method for deep learning-based biomedical image segmentation. Nat Methods. (2021) 18(2):203–11. 10.1038/s41592-020-01008-z 33288961

[12] Kirillov A Mintun E Ravi N Mao H Rolland C Gustafson L Segment Anything. *arXiv*. (2023). Available at: http://arxiv.org/abs/2304.02643 (Cited May 30, 2023).

[13] Ronneberger O Fischer P Brox T. U-Net: convolutional networks for biomedical image segmentation. In: Navab N Hornegger J Wells WM Frangi AF, editors. Medical image computing and computer-assisted intervention—MICCAI 2015. Cham: Springer International Publishing (2015). 234–41. p. (Lecture Notes in Computer Science).

[14] Muramatsu C Nishio M Goto T Oiwa M Morita T Yakami M Improving breast mass classification by shared data with domain transformation using a generative adversarial network. Comput Biol Med. (2020) 119:103698. 10.1016/j.compbiomed.2020.103698 32339129

[15] Anari PY Lay N Chaurasia A Gopal N Samimi S Harmon S Automatic segmentation of clear cell renal cell tumors, kidney, and cysts in patients with von Hippel-Lindau syndrome using U-net architecture on magnetic resonance images. *ArXiv*. (2023). arXiv:2301.02538v1.

[16] Rauschecker AM Gleason TJ Nedelec P Duong MT Weiss DA Calabrese E Interinstitutional portability of a deep learning brain MRI lesion segmentation algorithm. Radiol Artif Intell. (2022) 4(1):e200152. 10.1148/ryai.2021200152 35146430 PMC8823451

[17] Moazami S Ray D Pelletier D Oberai AA. Probabilistic brain extraction in MR images via conditional generative adversarial networks. bioRxiv. (2022). Available at: https://www.biorxiv.org/content/10.1101/2022.03.14.484346v1 (Cited Jun 16, 2023). 2022.03.14.484346 10.1109/TMI.2023.3327942 37883281

[18] Langlotz CP Allen B Erickson BJ Kalpathy-Cramer J Bigelow K Cook TS A roadmap for foundational research on artificial intelligence in medical imaging: from the 2018 NIH/RSNA/ACR/the academy workshop. Radiology. (2019) 291(3):781–91. 10.1148/radiol.2019190613 30990384 PMC6542624

[19] Antonelli M Reinke A Bakas S Farahani K Kopp-Schneider A Landman BA The medical segmentation decathlon. Nat Commun. (2022) 13(1):4128. 10.1038/s41467-022-30695-9 35840566 PMC9287542

[20] Mei X Liu Z Robson PM Marinelli B Huang M Doshi A Radimagenet: an open radiologic deep learning research dataset for effective transfer learning. Radiol Artif Intell. (2022) 4(5):e210315. 10.1148/ryai.210315 36204533 PMC9530758

[21] Weinstein JN Collisson EA Mills GB Shaw KM Ozenberger BA Ellrott K The cancer genome atlas pan-cancer analysis project. Nat Genet. (2013) 45(10):1113–20. 10.1038/ng.2764 24071849 PMC3919969

[22] Yan K Wang X Lu L Summers RM. Deeplesion: automated mining of large-scale lesion annotations and universal lesion detection with deep learning. J Med Imaging. (2018) 5(3):036501. 10.1117/1.JMI.5.3.036501 PMC6052252 30035154

[23] Karimi D Warfield SK Gholipour A. Transfer learning in medical image segmentation: new insights from analysis of the dynamics of model parameters and learned representations. Artif Intell Med. (2021) 116:102078. 10.1016/j.artmed.2021.102078 34020754 PMC8164174

[24] Jeong JJ Tariq A Adejumo T Trivedi H Gichoya JW Banerjee I. Systematic review of generative adversarial networks (GANs) for medical image classification and segmentation. J Digit Imaging. (2022) 35(2):137–52. 10.1007/s10278-021-00556-w 35022924 PMC8921387

[25] Skandarani Y Jodoin PM Lalande A. GANs for medical image synthesis: an empirical study. J Imaging. (2023) 9(3):69. 10.3390/jimaging9030069 36976120 PMC10055771

[26] Nishio M Fujimoto K Matsuo H Muramatsu C Sakamoto R Fujita H. Lung cancer segmentation with transfer learning: usefulness of a pretrained model constructed from an artificial dataset generated using a generative adversarial network. Front Artif Intell. (2021) 4:694815. 10.3389/frai.2021.694815 34337394 PMC8322116

[27] Shorten C Khoshgoftaar TM. A survey on image data augmentation for deep learning. J Big Data. (2019) 6(1):60. 10.1186/s40537-019-0197-0 PMC8287113 34306963

[28] Covidence systematic review software, Veritas Health Innovation, Melbourne, Australia. Available at: [www.covidence.org](http://www.covidence.org/).

[29] Baidya Kayal E Kandasamy D Sharma R Bakhshi S Mehndiratta A. Segmentation of osteosarcoma tumor using diffusion weighted MRI: a comparative study using nine segmentation algorithms. Signal Image Video Process. (2020) 14(4):727–35. 10.1007/s11760-019-01599-x

[30] Chang CY Buckless C Yeh KJ Torriani M. Automated detection and segmentation of sclerotic spinal lesions on body CTs using a deep convolutional neural network. Skelet Radiol. (2022) 51(2):391–9. 10.1007/s00256-021-03873-x 34291325

[31] Chang CY Huber FA Yeh KJ Buckless C Torriani M. Original research: utilization of a convolutional neural network for automated detection of lytic spinal lesions on body CTs. Skelet Radiol. (2023) 52:1377–84. 10.1007/s00256-023-04283-x 36651936

[32] Chen X Ma X Yan X Luo F Yang S Wang Z Personalized auto-segmentation for magnetic resonance imaging–guided adaptive radiotherapy of prostate cancer. Med Phys. (2022) 49(8):4971–9. 10.1002/mp.15793 35670079

[33] Chmelik J Jakubicek R Walek P Jan J Ourednicek P Lambert L Deep convolutional neural network-based segmentation and classification of difficult to define metastatic spinal lesions in 3D CT data. Med Image Anal. (2018) 49:76–88. 10.1016/j.media.2018.07.008 30114549

[34] Ding Y Chen Z Wang Z Wang X Hu D Ma P Three-dimensional deep neural network for automatic delineation of cervical cancer in planning computed tomography images. J Appl Clin Med Phys. (2022) 23(4):e13566. 10.1002/acm2.13566 35192243 PMC8992957

[35] Faghani S Baffour FI Ringler MD Hamilton-Cave M Rouzrokh P Moassefi M A deep learning algorithm for detecting lytic bone lesions of multiple myeloma on CT. Skelet Radiol. (2023) 52(1):91–8. 10.1007/s00256-022-04160-z 35980454

[36] Fan X Zhang X Zhang Z Jiang Y. Deep learning-based identification of spinal metastasis in lung cancer using spectral CT images. Sci Program. (2021) Article ID 2779390, 7. 10.1155/2021/2779390

[37] Fan X Zhang X Zhang Z Jiang Y. Deep learning on MRI images for diagnosis of lung cancer spinal bone metastasis. Contrast Media Mol Imaging. (2021) 2021:5294379. 10.1155/2021/5294379 34354553 PMC8294999

[38] Huang L Xia W Zhang B Qiu B Gao X. MSFCN-multiple supervised fully convolutional networks for the osteosarcoma segmentation of CT images. Comput Meth Prog Bio. (2017) 143:67–74. 10.1016/j.cmpb.2017.02.013 28391820

[39] Huo T Xie Y Fang Y Wang Z Liu P Duan Y Deep learning-based algorithm improves radiologists’ performance in lung cancer bone metastases detection on computed tomography. Front Oncol. (2023) 13:1125637. 10.3389/fonc.2023.1125637 36845701 PMC9946454

[40] Hwang D Kang SK Kim KY Seo S Paeng JC Lee DS Generation of PET attenuation map for whole-body time-of-flight (18)F-FDG PET/MRI using a deep neural network trained with simultaneously reconstructed activity and attenuation maps. J Nucl Med. (2019) 60(8):1183–9. 10.2967/jnumed.118.219493 30683763 PMC6681691

[41] Jin J Zhou H Sun S Tian Z Ren H Feng J Machine learning based gray-level co-occurrence matrix early warning system enables accurate detection of colorectal cancer pelvic bone metastases on MRI. Front Oncol. (2023) 13:1121594. 10.3389/fonc.2023.1121594 37035167 PMC10073745

[42] Johnsson K Brynolfsson J Sahlstedt H Nickols NG Rettig M Probst S Analytical performance of aPROMISE: automated anatomic contextualization, detection, and quantification of [(18)F]DCFPyL (PSMA) imaging for standardized reporting. Eur J Nucl Med Mol Imaging. (2022) 49(3):1041–51. 10.1007/s00259-021-05497-8 34463809 PMC8803714

[43] Ling Z Yang S Gou F Dai Z Wu J. Intelligent assistant diagnosis system of osteosarcoma MRI image based on transformer and convolution in developing countries. IEEE J Biomed Health Inform. (2022) 26(11):5563–74. 10.1109/JBHI.2022.3196043 35921344

[44] Lingappa E Parvathy LR. Deep learning-based active contour technique with bagging and boosting algorithms hybrid approach for detecting bone cancer from mri scan images. Multimed Tools Appl. (2023). 10.1007/s11042-023-14811-5

[45] Liu F Zhu J Lv B Yang L Sun W Dai Z Auxiliary segmentation method of osteosarcoma MRI image based on transformer and U-net. Comput Intell Neurosci. (2022) 2022:9990092. 10.1155/2022/9990092 36419505 PMC9678467

[46] Liu F Gou F Wu J. An attention-preserving network-based method for assisted segmentation of osteosarcoma MRI images. Mathematics. (2022) 10:1665. 10.3390/math10101665

[47] Liu X Han C Cui Y Xie T Zhang X Wang X. Detection and segmentation of pelvic bones metastases in MRI images for patients with prostate cancer based on deep learning. Front Oncol. (2021) 11:773299. 10.3389/fonc.2021.773299 34912716 PMC8666439

[48] Liu X Han C Wang H Wu J Cui Y Zhang X Fully automated pelvic bone segmentation in multiparameteric MRI using a 3D convolutional neural network. Insights Imaging. (2021) 12(1):93. 10.1186/s13244-021-01044-z 34232404 PMC8263843

[49] Lv B Liu F Li Y Nie J Gou F Wu J. Artificial intelligence-aided diagnosis solution by enhancing the edge features of medical images. Diagnostics. (2023) 13(6):1063. 10.3390/diagnostics13061063 36980371 PMC10047640

[50] Moreau N Rousseau C Fourcade C Santini G Ferrer L Lacombe M Deep learning approaches for bone and bone lesion segmentation on 18FDG PET/CT imaging in the context of metastatic breast cancer(). Annu Int Conf IEEE Eng Med Biol Soc. (2020) 2020:1532–5. 10.1109/EMBC44109.2020.9175904 33018283

[51] Moreau N Rousseau C Fourcade C Santini G Ferrer L Lacombe M Influence of inputs for bone lesion segmentation in longitudinal (18)F-FDG PET/CT imaging studies. Annu Int Conf IEEE Eng Med Biol Soc. (2022) 2022:4736–9. 10.1109/EMBC48229.2022.9871081 36086627

[52] Ouyang T Yang S Gou F Dai Z Wu J. Rethinking U-net from an attention perspective with transformers for osteosarcoma MRI image segmentation. Comput Intell Neurosci. (2022) 2022:7973404. 10.1155/2022/7973404 35707196 PMC9192230

[53] Qu Y Li X Yan Z Zhao L Zhang L Liu C Surgical planning of pelvic tumor using multi-view CNN with relation-context representation learning. Med Image Anal. (2021) 69:101954. 10.1016/j.media.2020.101954 33550006

[54] Schott B Weisman AJ Perk TG Roth AR Liu G Jeraj R. Comparison of automated full-body bone metastases delineation methods and their corresponding prognostic power. Phys Med Biol. (2023) 68(3):035011. 10.1088/1361-6560/acaf22 36580684

[55] Shen Y Gou F Dai Z. Osteosarcoma MRI image-assisted segmentation system base on guided aggregated bilateral network. Mathematics. (2022) 10(7):1090. 10.3390/math10071090

[56] Shuai L Zou W Hu N Gao X Wang J. An advanced W-shaped network with adaptive multi-scale supervision for osteosarcoma segmentation. Bio Signal Process Control. (2023) 80:104243. 10.1016/j.bspc.2022.104243

[57] Trägårdh E Enqvist O Ulén J Jögi J Bitzén U Hedeer F Freely available, fully automated AI-based analysis of primary tumour and metastases of prostate cancer in whole-body [(18)F]-PSMA-1007 PET-CT. Diagnostics. (2022) 12:2101. 10.3390/diagnostics12092101 36140502 PMC9497460

[58] Wang J Shi X Yao X Ren J Du X. Deep learning-based CT imaging in diagnosing myeloma and its prognosis evaluation. J Heal Eng. (2021) 2021:5436793. 10.1155/2021/5436793 PMC8452442 34552707

[59] Wang L Yu L Zhu J Tang H Gou F Wu J. Auxiliary segmentation method of osteosarcoma in MRI images based on denoising and local enhancement. Healthcare. (2022) 10:1468. 10.3390/healthcare10081468 36011123 PMC9408522

[60] Wu J Guo Y Gou F Dai Z. A medical assistant segmentation method for MRI images of osteosarcoma based on DecoupleSegNet. Int J Intell Syst. (2022) 37(11):8436–61. 10.1002/int.22949

[61] Wu J Liu Z Gou F Zhu J Tang H Zhou X BA-GCA Net: boundary-aware grid contextual attention net in osteosarcoma MRI image segmentation. Comput Intell Neurosci. (2022) 2022:3881833. 10.1155/2022/3881833 35942441 PMC9356797

[62] Wu J Xiao P Huang H Gou F Zhou Z Dai Z. An artificial intelligence multiprocessing scheme for the diagnosis of osteosarcoma MRI images. IEEE J Biomed Health Inf. (2022) 26(9):4656–67. 10.1109/JBHI.2022.3184930 35727772

[63] Wu J Yang S Gou F Zhou Z Xie P Xu N Intelligent segmentation medical assistance system for MRI images of osteosarcoma in developing countries. Comput Math Methods Med. (2022) 2022:7703583. 10.1155/2022/7703583 35096135 PMC8791734

[64] Wu J Zhou L Gou F Tan Y. A residual fusion network for osteosarcoma MRI image segmentation in developing countries. Comput Intell Neurosci. (2022) 2022:7285600. 10.1155/2022/7285600 35965771 PMC9365532

[65] Xu L Tetteh G Lipkova J Zhao Y Li H Christ P Automated whole-body bone lesion detection for multiple myeloma on (68)Ga-pentixafor PET/CT imaging using deep learning methods. Contrast Media Mol Imaging. (2018) 2018:2391925. 10.1155/2018/2391925 29531504 PMC5817261

[66] Yildiz Potter I Yeritsyan D Mahar S Wu J Nazarian A Vaziri A. Automated bone tumor segmentation and classification as benign or malignant using computed tomographic imaging. J Digit Imaging. (2023) 36:869–78. 10.1007/s10278-022-00771-z 36627518 PMC10287871

[67] Zhan X Liu J Long H Zhu J Tang H Gou F An intelligent auxiliary framework for bone malignant tumor lesion segmentation in medical image analysis. Diagnostics. (2023) 13(2):223. 10.3390/diagnostics13020223 36673032 PMC9858155

[68] Zhang R Huang L Xia W Zhang B Qiu B Gao X. Multiple supervised residual network for osteosarcoma segmentation in CT images. Comput Med Imaging Graph. (2018) 63:1–8. 10.1016/j.compmedimag.2018.01.006 29361340

[69] Zhao Y Gafita A Vollnberg B Tetteh G Haupt F Afshar-Oromieh A Deep neural network for automatic characterization of lesions on (68)Ga-PSMA-11 PET/CT. Eur J Nucl Med Mol Imaging. (2020) 47(3):603–13. 10.1007/s00259-019-04606-y 31813050

[70] Paranavithana IR Stirling D Ros M Field M. Systematic review of tumor segmentation strategies for bone metastases. Cancers (Basel). (2023) 15(6):1750. 10.3390/cancers15061750 36980636 PMC10046265

[71] Faiella E Santucci D Calabrese A Russo F Vadalà G Zobel BB Artificial intelligence in bone metastases: an MRI and CT imaging review. Int J Environ Res Public Health. (2022) 19(3):1880. 10.3390/ijerph19031880 35162902 PMC8834956

[72] Yang C Qin LH Xie YE Liao JY. Deep learning in CT image segmentation of cervical cancer: a systematic review and meta-analysis. Radiat Oncol. (2022) 17(1):175. 10.1186/s13014-022-02148-6 36344989 PMC9641941

[73] Carvalho LE Sobieranski AC von Wangenheim A. 3D Segmentation algorithms for computerized tomographic imaging: a systematic literature review. J Digit Imaging. (2018) 31(6):799–850. 10.1007/s10278-018-0101-z 29915942 PMC6261188

[74] Domingues I Pereira G Martins P Duarte H Santos J Abreu PH. Using deep learning techniques in medical imaging: a systematic review of applications on CT and PET. Artif Intell Rev. (2020) 53(6):4093–160. 10.1007/s10462-019-09788-3

[75] Akkus Z Galimzianova A Hoogi A Rubin DL Erickson BJ. Deep learning for brain MRI segmentation: state of the art and future directions. J Digit Imaging. (2017) 30(4):449–59. 10.1007/s10278-017-9983-4 28577131 PMC5537095

[76] Shal K Choudhry MS. Evolution of deep learning algorithms for MRI-based brain tumor image segmentation. Crit Rev Biomed Eng. (2021) 49(1):77–94. 10.1615/CritRevBiomedEng.2021035557 34347989

[77] Gul S Khan MS Bibi A Khandakar A Ayari MA Chowdhury MEH. Deep learning techniques for liver and liver tumor segmentation: a review. Comput Biol Med. (2022) 147:105620. 10.1016/j.compbiomed.2022.105620 35667155

[78] Fleiss J. The measurement of interrater agreement. In: Statistical methods for rates and proportions. 2nd ed. New York: John Wiley & Sons (1981). 212–36.

[79] Taha AA Hanbury A. Metrics for evaluating 3D medical image segmentation: analysis, selection, and tool. BMC Med Imaging. (2015) 15(1):29. 10.1186/s12880-015-0068-x 26263899 PMC4533825

[80] Zijdenbos AP Dawant BM Margolin RA Palmer AC. Morphometric analysis of white matter lesions in MR images: method and validation. IEEE Trans Med Imaging. (1994) 13(4):716–24. 10.1109/42.363096 18218550

[81] Zou KH Warfield SK Bharatha A Tempany CMC Kaus MR Haker SJ Statistical validation of image segmentation quality based on a spatial overlap index. Acad Radiol. (2004) 11(2):178–89. 10.1016/S1076-6332(03)00671-8 14974593 PMC1415224

[82] Alessio AM Kinahan PE Cheng PM Vesselle H Karp JS. PET/CT scanner instrumentation, challenges, and solutions. Radiol Clin North Am. (2004) 42(6):1017–32. 10.1016/j.rcl.2004.08.001 15488555

[83] Lin TY Maire M Belongie S Bourdev L Girshick R Hays J Microsoft COCO: Common Objects in Context. *arXiv*. (2015). Available at: http://arxiv.org/abs/1405.0312 (Cited Jun 11, 2023).

[84] Yin XX Sun L Fu Y Lu R Zhang Y. U-Net-Based medical image segmentation. J Healthc Eng. (2022) 2022:e4189781. 10.1155/2022/4189781 PMC9033381 35463660

[85] Carrino JA. An artificially intelligent solution for a real problem in musculoskeletal radiology: bone tumors. Radiology. (2021) 301(2):407–8. 10.1148/radiol.2021211560 34491136

[86] Von Schacky CE Wilhelm NJ Schäfer VS Leonhardt Y Gassert FG Foreman SC Multitask deep learning for segmentation and classification of primary bone tumors on radiographs. Radiology. (2021) 301(2):398–406. 10.1148/radiol.2021204531 34491126

[87] Zbinden L Doorenbos L Pissas T Huber AT Sznitman R Márquez-Neila P. Stochastic Segmentation with Conditional Categorical Diffusion Models. *arXiv*. (2023). Available at: http://arxiv.org/abs/2303.08888 (Cited Jun 16, 2023).

