# Supplementary material for: Deep learning image segmentation approaches for malignant bone lesions: a systematic review and meta-analysis
Source: Front Radiol. 2023 Aug 8;3:1241651. doi: 10.3389/fradi.2023.1241651 (PMC10442705; doi:10.3389/fradi.2023.1241651)
Supplement: Supplementary file 2 [file Datasheet1.docx]

Supplementary Material

Deep Learning Image Segmentation Approaches for Cancerous Lesions on CT and MRI Scans of the Bone

Joseph M. Rich^1*^, Lokesh Bhardwaj^1^, Aman Shah^2^, Krish Gangal^3^, Mohitha Rapaka^4^, Assad Oberai^5^, Brandon K.K. Fields^6^, George R. Matcuk Jr.^7^, Vinay A. Duddalwar^8,9^

*** Correspondence:** Corresponding Author: jmrich@usc.edu

# Supplementary Figures and Tables

Supplementary Table 1: Detailed characteristics of included studies

See Supplementary Table 1.xlsx

Supplementary Table 2: Median Dice similarity coefficients within different categories

| Categories | DSC | Interquartile Range |
| --- | --- | --- |
| All modalities / 2D | 0.912 | 0.230 |
| All modalities / 3D | 0.850 | 0.184 |
| NR Imaging Dimensionality | 0.912 | 0.063 |
| 2017 / Other Dimensionality | 0.903 | 0 |
| 2018 / 3D | 0.893 | 0 |
| 2019 / 3D | 0.770 | 0 |
| 2020 / 3D | 0.835 | 0.210 |
| 2020 / 2D | 0.600 | 0 |
| 2021 / Other Dimensionality | 0.888 | 0 |
| 2021 / 3D | 0.859 | 0.038 |
| 2022 / Other Dimensionality | 0.924 | 0.006 |
| 2022 / 3D | 0.882 | 0.340 |
| 2022 / 2D | 0.923 | 0.130 |
| 2023 / Other Dimensionality | 0.819 | 0 |
| 2023 / 3D | 0.725 | 0.190 |
| 2023 / 2D | 0.930 | 0.389 |
| CT / Other Dimensionality | 0.861 | 0.084 |
| CT / 3D | 0.856 | 0.101 |
| CT / 2D | 0.940 | 0.255 |
| CT / All Dimensionalities | 0.923 | 0.141 |
| MRI / Other Dimensionality | 0.921 | 0.039 |
| MRI / 3D | 0.876 | 0.089 |
| MRI / 2D | 0.912 | 0.053 |
| MRI / All Dimensionalities | 0.850 | 0.077 |
| PET-CT / 3D | 0.640 | 0.210 |
| PET-CT / 2D | 0.600 | 0 |
| PET-MRI / 3D | 0.770 | 0 |
| PET-CT, PET-MRI / All Dimensionalities | 0.903 | 0.210 |
| Blastic / 3D | 0.800 | 0.086 |
| Blastic / 2D | 0.895 | 0.260 |
| Lytic / 3D | 0.922 | 0.081 |
| Lytic / 2D | 0.940 | 0.200 |
| Other Tumor Type / 3D | 0.810 | 0.339 |
| Other Tumor Type / 2D | 0.901 | 0.364 |
| Other Tumor Type / Other Dimensionality | 0.903 | 0.033 |

DSC, Dice similarity coefficient; 2D, 2-dimensional; 3D, 3-dimensional; NR, not reported
